# MiRNA Analysis by Quantitative PCR in Preterm Human Breast Milk Reveals Daily Fluctuations of hsa-miR-16-5p

**DOI:** 10.1371/journal.pone.0140488

**Published:** 2015-10-16

**Authors:** Ilaria Floris, Hélène Billard, Clair-Yves Boquien, Evelyne Joram-Gauvard, Laure Simon, Arnaud Legrand, Cécile Boscher, Jean-Christophe Rozé, Francisco Bolaños-Jiménez, Bertrand Kaeffer

**Affiliations:** 1 UMR-1280, INRA, University of Nantes, Physiologie des Adaptations Nutritionnelles, Nantes, France; 2 University of Nantes, Hospital of Mother and Child, Nantes, France; University of Florida, UNITED STATES

## Abstract

**Background and Aims:**

Human breast milk is an extremely dynamic fluid containing many biologically-active components which change throughout the feeding period and throughout the day. We designed a miRNA assay on minimized amounts of raw milk obtained from mothers of preterm infants. We investigated changes in miRNA expression within month 2 of lactation and then over the course of 24 hours.

**Materials and Methods:**

Analyses were performed on pooled breast milk, made by combining samples collected at different clock times from the same mother donor, along with time series collected over 24 hours from four unsynchronized mothers. Whole milk, lipids or skim milk fractions were processed and analyzed by qPCR. We measured hsa-miR-16-5p, hsa-miR-21-5p, hsa-miR-146-5p, and hsa-let-7a, d and g (all -5p). Stability of miRNA endogenous controls was evaluated using RefFinder, a web tool integrating geNorm, Normfinder, BestKeeper and the comparative ΔΔCt method.

**Results:**

MiR-21 and miR-16 were stably expressed in whole milk collected within month 2 of lactation from four mothers. Analysis of lipids and skim milk revealed that miR-146b and let-7d were better references in both fractions. Time series (5H-23H) allowed the identification of a set of three endogenous reference genes (hsa-let-7d, hsa-let-7g and miR-146b) to normalize raw quantification cycle (Cq) data. We identified a daily oscillation of miR-16-5p.

**Perspectives:**

Our assay allows exploring miRNA levels of breast milk from mother with preterm baby collected in time series over 48–72 hours.

## Introduction

MicroRNAs (miRNAs) are short noncoding RNA sequences that regulate gene expression by inhibiting mRNA translation into protein [1]. MiRNAs are present in many body fluids, including breast milk, amniotic fluid, saliva, urine [2]. Particular attention is paid to amniotic fluid and breast milk because, among all biological fluids, the first is provided to the fetus and the second, orally, to the newborn. Both amniotic fluid and milk have nutritive, protective and regulatory roles [3]. MiRNAs are recognized as important players of many biological processes in fetal life (organogenesis and morphogenesis) [4]. Studies in human breast milk have identified more than 600 miRNAs with known functions: many have immune-related functions (regulatory effect on T-cells, induction of B-cell differentiation); whilst others may regulate physiological and metabolic responses [5]. Remarkably, there is some evidence that biochemical signals occurring via amniotic fluid *in utero*, show continuity after birth *via milk* [6].

In milk miRNAs are stable, in spite of the high content of RNases which are able to neutralize viral and bacterial nucleic acids [7, 8], because they are sheltered inside milk extracellular vesicles [9], milk fat globules [10,11] and cellular components [12]. MiRNAs in milk remain stable after incubation at room temperature, multiple freeze-thaw cycles, treatment with RNases, and incubation at 100°C for 10 minutes [13]. Moreover, they are resistant to acidic environments [13, 14].

Two approaches have been developed to address the challenge of accurate miRNA analysis by qPCR: (1) stem-loop miRNA-specific reverse transcription primers, followed by miRNA-specific probe detection [15], or (2) homopolymeric tails non-specific for all miRNAs followed by intercalating dye-based detection [16]. Here we have used the stem-loop miRNA-specific reverse transcription primers followed by specific TaqMan probe-based target detection.

We propose a miRNA assay using minimized amounts of raw milk. We have tested the stability of six candidate endogenous controls (ECs), with selection based on the literature: hsa-miR-146b-5p [5, 10, 14], hsa-miR-16-5p [17, 18], hsa-miR-21-5p [17], hsa-let-7a-5p [18], hsa-let-7g-5p [19], and hsa-let-7d-5p [18–20]. Of these, miR-16-5p has been used as an EC in human breast milk [17] and hsa-miR-146b-5p is one of the most abundant miRNAs in human milk [10, 14].

Taking into consideration the extreme complexity of human milk, which contains many biologically active components (cytokines, hormones, nucleotides) that change during the course of the feeding period and throughout the day [21–23], we further decided to proceed in two steps. Firstly, we have used pooled breast milk, combining samples collected at different clock times during a single day of lactation from a single mother donor, in order to ‘mask’ the daily fluctuations. We could observe miRNA variations during lactation only. The second set of samples has been examined to identify miRNA fluctuations over the course of 24 hours.

In this paper, we propose a set of endogenous reference genes (hsa-let-7d-5p, hsa-let7g-5p, and hsa-miR-146b-5p) to normalize raw quantification cycle (Cq) data and we identified a daily fluctuation of miR-16-5p in preterm milk.

## Materials and Methods

### Ethics statement

All mothers involved in the study signed informed consent forms to participate. The study was approved by the Hospital Ethics Committees at the Hospital of Mother and Child, Nantes (France). The protocol was approved by the Nantes Hospital Ethics Committee as Lactacol NCT01493063 under the guidance of Dr Cécile Boscher as ancillary study.

### Milk sample collection

Human mature milk samples (Lactacol NCT01493063) were collected by healthy mothers that gave birth to preterm babies (84 milk samples from 22 mothers). The samples were transported to the laboratory and stored at -80°C until further analysis. Fresh milk samples (2 donors) were used within 20 minutes of collection; an aliquot was stored frozen at -80°C for 24 hours and another for 1 month, until further analysis. Most of the collected sample volumes for q-PCR were of 100 μL.

### Milk samples: lipids and skim milk separation

Human breast milk (from 50, 100 or 300 μL) was transferred into sterile, RNase-free tubes and centrifuged at 800xg for 10 min at 4°C. The centrifugation separated the milk into three fractions: the upper layer of fat globules, the skim milk and the cellular pellet. Using a needle from a 0.5ml syringe (Terumo, Myjector U-100), the pellet was first detached from the tube, and then removed together with the skim milk by aspiration, leaving just the lipids on the tube wall for further RNA extraction.

The aspirated skim milk underwent a second centrifugation at 800xg for 10 min at 4°C. The resulting skim milk was aspirated using a 0.5mL syringe, leaving the cellular pellet, which was transferred into a new tube and used for RNA extraction.

### RNA extraction

One ml of Qiazol (Qiagen) was added to whole milk (50, 100, 300 μL), to lipids or to skim milk fractions. To ensure effective denaturation, the samples were well mixed by vortexing and incubated for 5 min at room temperature.

The addition of 0.2 volumes of chloroform allows aqueous and organic phase separation. To obtain a clear aqueous phase the samples were vortexed at the maximum setting for 30 sec, and then centrifuged at 12,000xg for 15 min at 4°C. The aqueous phase was carefully transferred to a new tube. Subsequently, 1 ml of isopropanol was added and the samples incubated for 10 min at room temperature. To improve yield, the RNA was precipitated at -20°C overnight. Centrifugation at 12,000xg for 15 min at 4°C pelleted the RNA, which was then washed in 70% ethanol and resuspended in nuclease-free water (15 μL). The RNA concentrations were assessed by spectrophotometry using Nanodrop (Thermo Scientific).

### Quality of RNA preparations

A260/A280 ratios were analyzed by Nanodrop to assess the RNA purity [24]. However, because many RT-qPCR inhibitors are not detected by spectrophotometric analysis [24], we performed a *spike assay* to identify the presence of qPCR inhibitors.

We used *cel-lin4-5p*, a miRNA from *C*. *elegans*, as a spike control. An aliquot (280 μg) was dissolved in nuclease free water to a concentration of 100 pmol/μl; then working stocks were prepared at 200 nM (0.5 ng/μL). A standard curve was made using serial dilutions of the spike-in with water (S1 Fig). To assess reaction inhibition, the spike control (1x10^-5^ ng) was added (1) to the experimental RNA sample and, in parallel, (2) to a water sample and (3) to 3% phenol. The spike control was reverse transcribed and the resulting cDNA was subsequently amplified using a spike primer assay (Applied Biosystems). ΔCq values [Cq (spike control in RNA)—Cq (spike control in water)] were measured as an indicator of the presence of reaction inhibitors. ΔCq values < 2 indicate no significant reaction inhibition, values between 2 and 3 suggest likely reaction inhibition, and values > 3 indicate strong reaction inhibition.

The RNA samples checked for purity were spike-free, meaning that no cel-lin4-5p was added before RNA extraction. All the RNA samples tested showed ΔCq values < 1, suggesting that no inhibition was affecting the RT-qPCR results, while 3% phenol resulted in strong inhibition with ΔCq > 4.

### Reverse transcription

The TaqMan miRNA Reverse Transcription Kit (Applied Biosystems, France) and miRNA-specific stem–loop primers (Applied Biosystems) were used for miRNA Reverse transcription (RT) in a scaled down RT reaction with a final volume of 5 μL [25]. All RNA samples were diluted using 2 μL from the stocks to obtain a working concentration of 2 ng/μL. Each reaction consisted of 2 μL RNA (around 4 ng) combined with 3 μL of master mix, prepared by using Applied Biosystems components (1.38 μL of nuclease-free H_2_O; 1 μL of TaqMan miRNA (5X) RT primer; 0.5 μL of 10X RT buffer; 0.063 μL of RNase inhibitor; 0.05 μL of 100 mmol/L deoxynucleoside triphosphates, and 0.3 μL of MultiScribe reverse transcriptase). For spike-in *C*. *elegans* cel-lin4-5p standard curves, serially diluted synthetic miRNAs were added to the RT reaction in parallel with experimental samples.

RT was carried out in a thermal cycler (CFX Connect™ Real-Time PCR Detection System) at 16°C for 30 min, 42°C for 30 min, and 85°C for 5 min, hold at 4°C and stored at -20°C prior to qPCR.

### Real Time PCR

MiRNAs were quantified by qPCR with TaqMan Fast Universal PCR Master Mix (Applied Biosystems) and individual specific miRNA primers and hydrolysis probes (Applied Biosystems). Each reaction (10 μL) has been made in duplicate combining 2 μL of RT product with 9 μl of nuclease-free H_2_O, 11.5 μl of TaqMan Fast Universal PCR Master Mix (2X), and 1 μL of TaqMan miRNA Assay (20X) primers. The TaqMan probe identifiers were as follows: hsa-miR-16-5p (#000391), hsa-let7g-5p (#002282), hsa-let-7a-5p (#000377), hsa-let-7d-5p (#002283), hsa-miR-146b-5p (#001097), hsa-miR-21-5p (#000397). Real time PCR was performed using the following conditions: 95°C for 10 min, followed by 40 cycles of 95°C for 15 s and 60°C for 1 min.

### Data and Statistical Analysis

The Stability of miRNA references was determined using RefFinder, software that integrates the currently available major computational programs (geNorm [26], Normfinder [27], BestKeeper [28], and the comparative ΔΔCt method [29]). Correlation and statistical analyses were achieved using GraphPad Prism (v5, GraphPad, California). MiRWalk [30] was utilized to scrutinize period1 and clock mRNA sequences for 3'UTR, 5'UTR and promoter target sites. We used RNAhybrid software [31] to find the *minimum free energy hybridization* (*mfe*) for miRNA-target prediction.

Comparisons between miRNA Cq data in whole milk, lipids and skim milk were performed using one-way ANOVA (with Tukey's *post hoc* multiple comparison test). We used the comparative 2^-ΔCq^ method to evaluate expression levels of miR-16, miR-21 and let-7a during clock time, using the geometric mean of let-7g and let-7d (let-7g/d) or the geometric mean of let-7g, let-7d and miR-146b (let-7g/d/miR146-b) as normalization factors. One-way ANOVA (with Tukey's *post hoc* multiple comparison test) was performed to assess miRNA variations throughout the 24 hours.

## Results

### RNA yield and concentration from whole preterm milk

Total RNA concentration, considerably higher than that of other fluids (e.g. serum) [2], allows the standardization of total RNA input for the reverse transcription reaction. We processed 50, 100 and 300 μL of whole milk (n = 2 each) and respectively got 4, 8 and 9 μg of total RNA. With the intention of purifying milk fractions, we tested our method using 50, 100 and 300 μL of whole milk. Using 300 and 100 μL we could see clearly, after centrifugation, the three fractions (lipids, skim milk and cellular pellet). The use of 50 μL as starting volume was insufficient to permit observation of the cellular pellet with the naked eye. The amount of RNA recovered from 300 μL was not optimized with higher amount of Qiazol. To minimize the amount of milk required for the analysis, 100 μL was preferred over 50 μL or 300 μL. In our clinical trial, we can obtain 300 μL routinely which means that miRNAs can be assayed both on crude and purified milk fractions leaving 100 μL for later controls. RNA extracted from whole milk samples (n = 55) gave concentrations ranging from 105 to 665 ng/μL (mean concentration = 313 ng/μL, SD = 110, CV = 35%; S2 Fig).

### MiRNAs tested in fresh and frozen human breast milk

Here, we tested whether frozen milk could affect miRNA levels measured in whole milk. S3 Fig shows the results of four independent experiments. No significant difference was found between fresh and stored milk, at least for miR-16, miR-21, let-7a, let-7g and let-7d. We also confirmed that miRNAs are stable after 1 month at -80°C.

### Comparison between external and endogenous miRNA controls

In order to assess the use of external or internal references, 21 milk samples were processed. External RNA (spike-in) cel-lin-4-5p was added before RNA extraction during the denaturation phase in Qiazol. As specified in the materials and methods, we prepared RNA dilutions (2 ng/μl) and input 4 ng for the RT reactions. The spike-in and the five selected endogenous miRNA references (miR-21-5p, miR-16-5p, let-7a-5p, let-7g-5p, let-7d-5p) were measured by real-time PCR. Detection of the external spike-in was relatively consistent revealing similar RNA efficiency between samples (mean Cq = 14.76, SD = 1.36, n = 21). However, when the endogenous references miR-16, miR-21 or let-7a were plotted against the external RNA cel-lin-4 for each sample, no correlation was found (S4A Fig). We plotted spike-in Cq values against the RNA dilution factors calculated to make the RNA dilutions(S4B Fig), and we observed a positive and significant correlation between spike-in Cq and RNA dilution factors (Pearson r = 0.51, *p = 0*.*016*). In line with this, spike-in expressional levels and RNA dilution factors were negatively correlated (r = -0.54, *p = 0*.*010*). It means that, when RNA has been highly diluted, spike-in expression was lower and vice versa. No correlation was found between RNA dilution factors and the five other ECs (data not shown). On the other hand, miR-16 and miR-21 Cq values, which are both candidate ECs, were both positively correlated (r = 0.58, *p = 0*.*006;* S4C Fig).

We conclude that spike-in external RNAs are a good measure of the RNA extraction efficiency, but they are not a suitable reference for miRNA normalization in milk. In fact, because of the high sample-to-sample variability in RNA concentration (S2 Fig) the differences in dilution factors to ensure the same RNA input caused differences in the distribution of external spike-in between samples.

### Candidate endogenous controls tested for stability within month 2 of lactation in human breast milk

**Fig 1:**
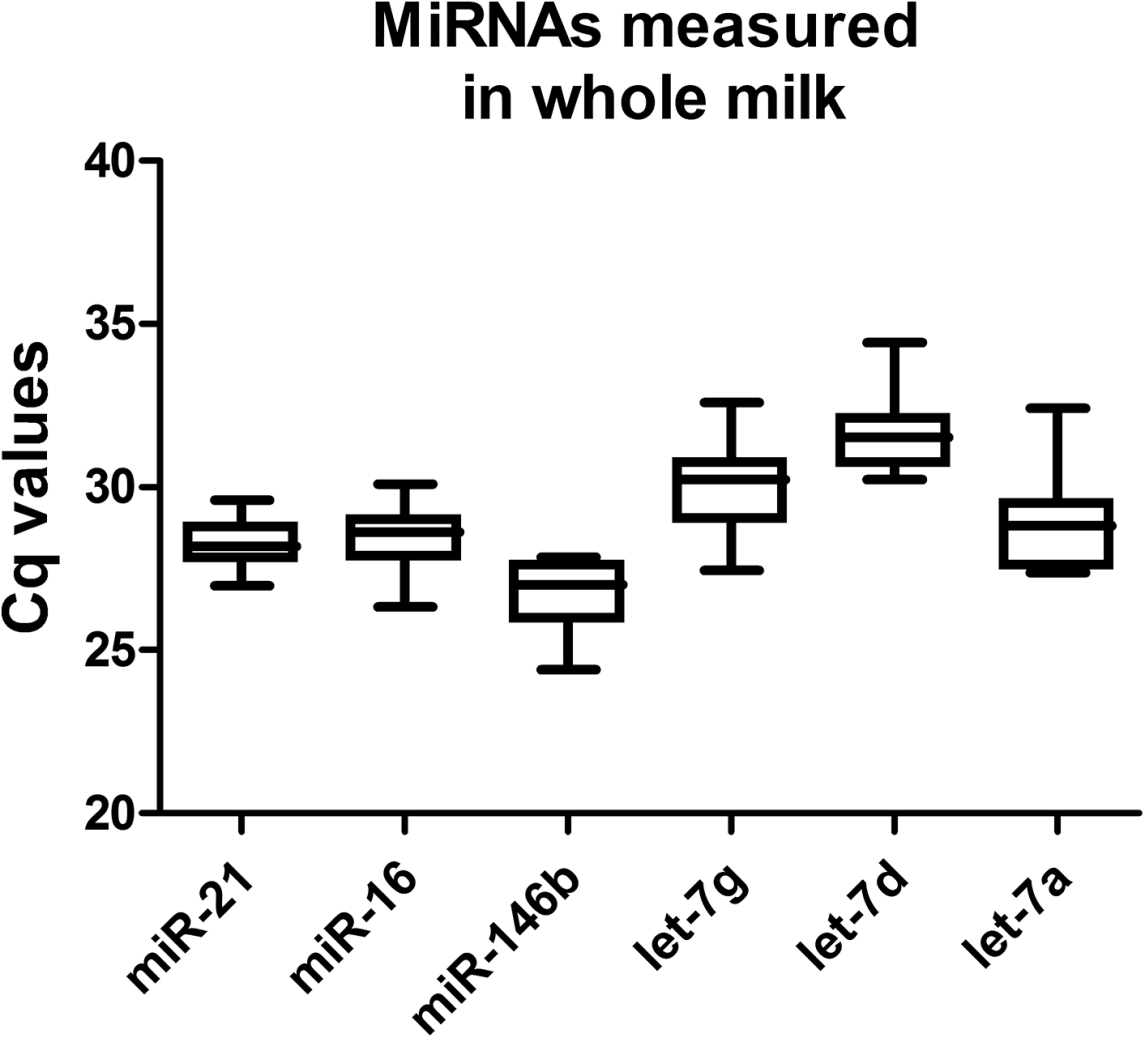
MiRNAs measured at different lactation periods in whole human preterm milk. Candidate endogenous references (let-7a, let-7g, let-7d, miR-16, miR-21 and miR-146b) were measured in mature human milk collected within month 2 of lactation, from 28 to 65 days postpartum. The Cq values of each miRNA are shown by box-and-whisker plots (1–99 percentile).

A simple and intuitive way to evaluate reference gene expression stability is the analysis of the variation; a higher variance is associated with less stability and poor normalization [32]. To determine the stability of six literature-based candidate miRNAs (miR-21, miR-16, let-7a, let-7g, let-7d) and miR-146b, we analyzed human breast milk (n = 15) collected within month 2 of lactation from four healthy mother donors (Table 1). Each sample represents a pool of milk collected during a single day of lactation from a single mother donor made by mixing equal quantities of breast milk collected from 2 to 5 time points during 24 hours. In that way, the clock time variations were masked in order to assess miRNA variations within month 2 of lactation. Raw Cq data of each candidate EC were grouped together and represented in box-and-whisker plots (1–99 percentile; Fig 1). These data revealed that miR-21 (mean Cq = 28.24, SD = 0.76) and miR-16 (mean Cq = 28.47, SD = 0.99) were highly stable during lactation in mature milk. MiR-146b was confirmed abundant and it also appeared relatively stable (mean Cq = 26.72, SD = 1.37).

**Table 1:** Milk samples used to analyze miRNA stability within month 2 of lactation.

Mother number	Body Mass Index	Lactation Period
		(days postpartum)
**1**	18	44
		51
		58
		65
**2**	23	30
		37
		41
		48
		57
		63
**3**	22	28
		35
		42
**4**	22	29
		36

### Candidate endogenous controls tested for stability in lipids and skim milk fractions

**Fig 2:**
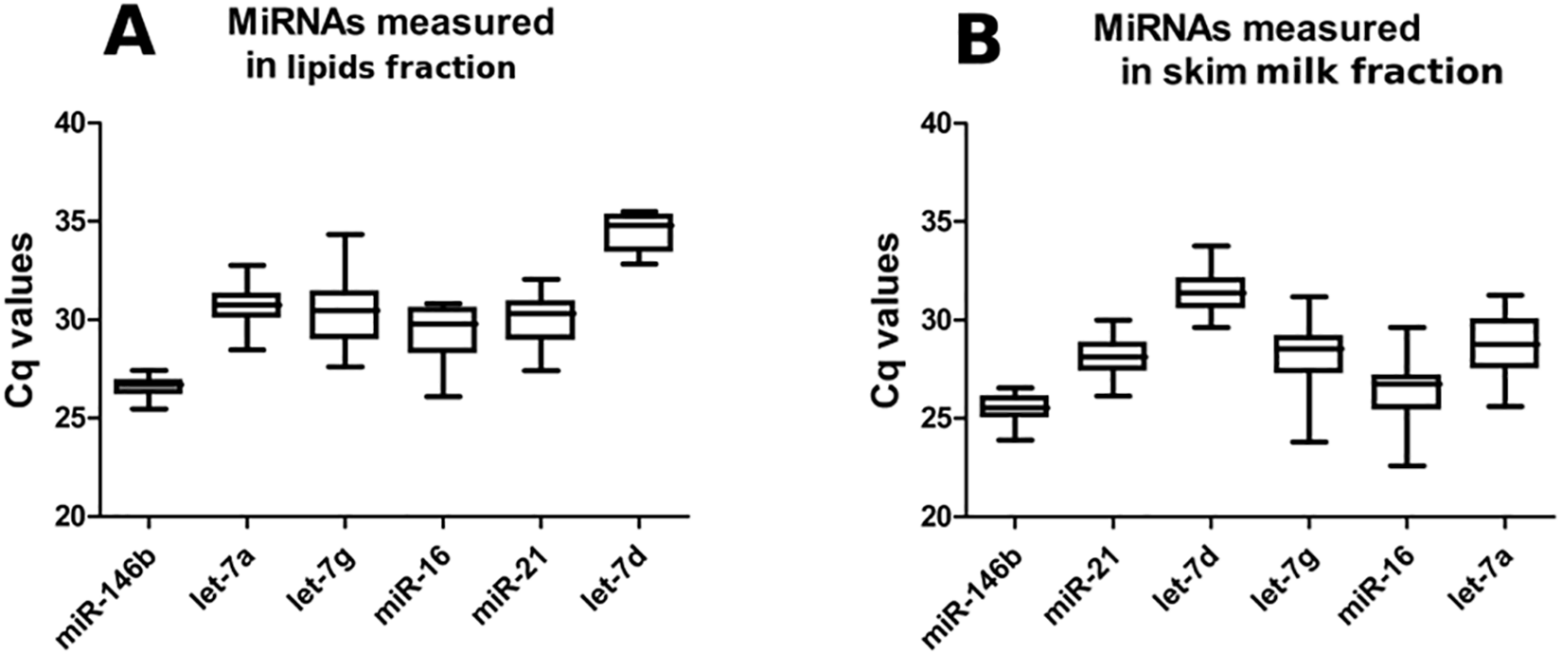
MiRNA levels measured in lipids and skim milk fractions at different lactation periods. Cq values by box-and-whisker plots (1–99 percentile) of endogenous references (let-7a, let-7g, let-7d, miR-16, miR-146b and miR-21) measured **A)** in lipids and **B)** in skim milk.

The same milk samples previously mentioned (Table 1) were purified for their lipids and skim milk fractions, as described in the materials and methods. Lipids and skim milk fractions respectively gave RNA concentrations ranging between 84.9–208.0 ng/μL (mean = 148 ng/μL, SD = 47.8 ng/μL, n = 15) and 103.1–264.0 ng/μL (mean = 161 ng/μL, SD = 41.4 ng/μL, n = 15). In addition, we tested the stability of the five miRNA controls and miR-146b within month 2 of lactation in lipids and skim milk. Raw Cq data of each miRNA were represented in a box-and-whisker plots (1–99 percentile) for both fractions (Fig 2A and 2B). Surprisingly, miR-146b was the most stable in lipids (mean Cq = 26.57, SD = 0.52) and in skim milk (mean Cq = 25.47, SD = 0.71). In skim milk, let-7d (mean Cq = 31.35, SD = 1.0) and miR-21 (mean Cq = 28.15, SD = 1.0) were also stable, while miR-16, let-7g and let-7a showed higher variability (Cq SD > 1.8). In lipids, let-7d (mean Cq = 34.37, SD = 0.92), let-7a (mean Cq = 30.69m SD = 1.0) and miR-21 (mean Cq = 29.9, SD = 1.26) were also stable, whilst miR-16 and let-7g showed higher variability (Cq SD > 1.4).

### Candidate endogenous controls checked for differential distribution between whole milk, lipids and skim milk fractions

**Fig 3:**
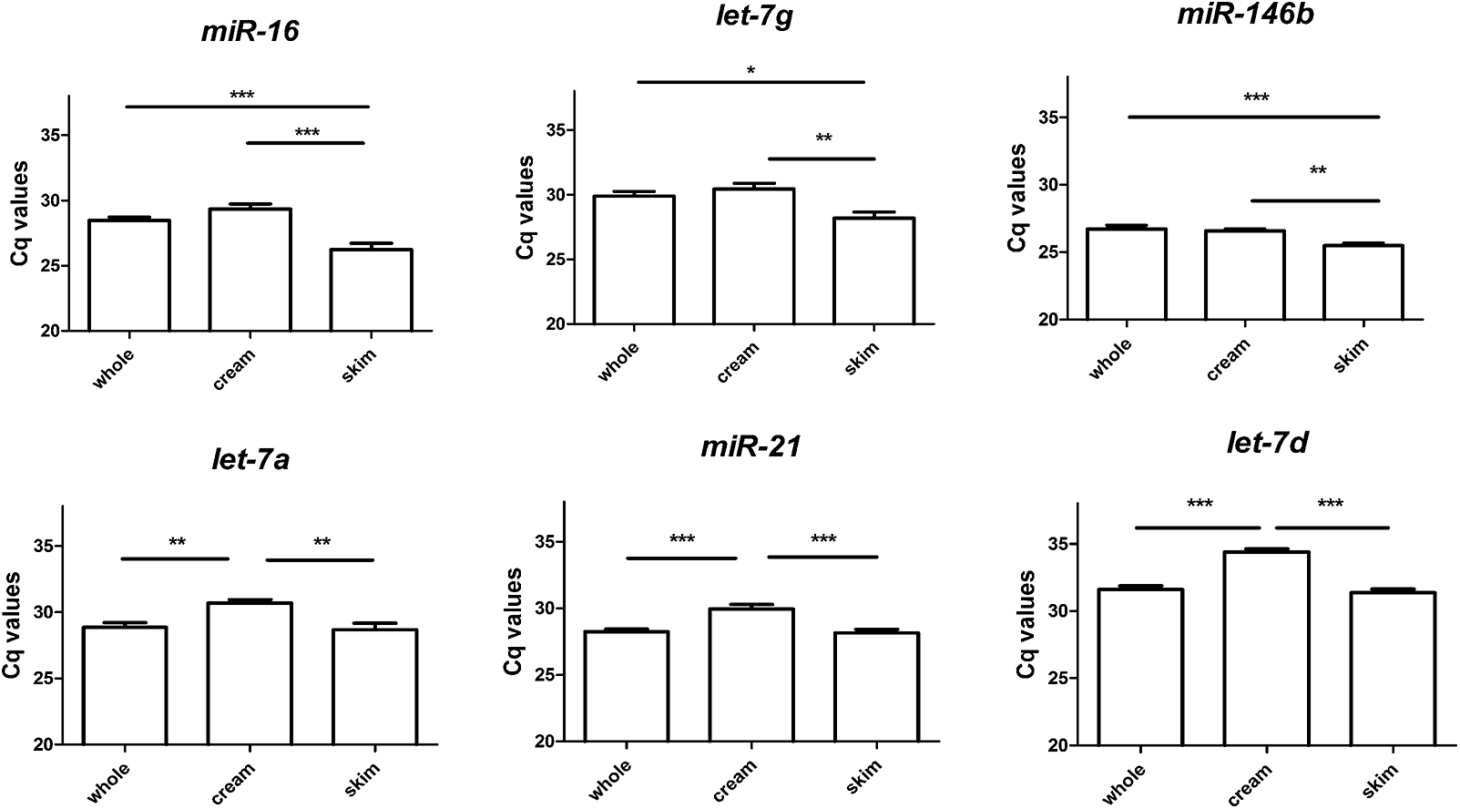
Differences in miRNA expression levels between whole milk, lipids and skim milk. Raw Cq data of endogenous references (let-7a, let-7g, let-7d, miR-16, miR-146b and miR-21) analyzed in full milk, lipids, and skim milk, were represented in a Tukey box plot. MiR-21, let-7a and let-7d expression was significantly lower in lipids than in skim and full milk, while no differences were found between skim milk and whole milk. MiR-16, let-7g and miR-146b expression was significantly higher in skim milk fractions than in lipids and in full milk (*p<0.05; **p<0.01; ***p<0.001).

We checked for differences in miRNA distribution between whole milk, lipids and skim milk. In Fig 3, we represented in a graph the mean Cq with SEM of the six measured miRNAs in whole milk or its fractions. MiR-21, let-7d and let-7a Cq values were significantly lower in lipids than in skim milk (respectively p<0.001, p<0.001 and *p<0*.*01*) and than in full milk (respectively *p<0*.*001*, *p<0*.*001* and *p<0*.*01*), whilst no differences were found between skim milk and whole milk. On the other hand, miR-16, let-7g and miR-146b Cq values were significantly higher in skim milk fractions than in lipids (respectively *p<0*.*001*, *p<0*.*01* and *p<0*.*01*) and whole milk (respectively *p<0*.*001*, *p<0*.*05* and *p<0*.*001*).

### Analysis of daily miRNA fluctuations in milk

In order to test miRNA stability throughout a single day of lactation and detect daily fluctuations, milk samples (n = 39) from four donor mothers, collected at different clock times, throughout 2 or 3 days (Table 2) were analyzed for miR-21-5p, miR-16-5p, let-7a-5p, let-7g-5p, let-7d-5p and miR-146b-5p. A miRWalk investigation allowed us to check potential miRNA binding sites with circadian core elements [30]. Often miRNAs which target core circadian genes show a 24-hour pattern too [33].

**Table 2:** Breast milk samples collected for testing daily miRNA fluctuations.

Mother name	Body Mass Index	Lactation Period(days postpartum)	Milk Collection Time (clock hours)					
**A**	33	30		07:30	11:00	17:30		21:00
		37	04:30	09:00		14:00	18:00	21:30
**B**	20	32		08:00	11:45	14:45	18:00	21:30
		42		06:45	09:15	15:00	18:00	
		45		08:00		13:45	17:30	23:00
**C**	20	49		07:30	10:30	15:00	19:00	
		56		07:15		14:00	17:15	22:15
**D**	20	31		07:00	12:10	15:30		22:30
		33		07:00	10:00	14:00	19:00	

**Fig 4:**
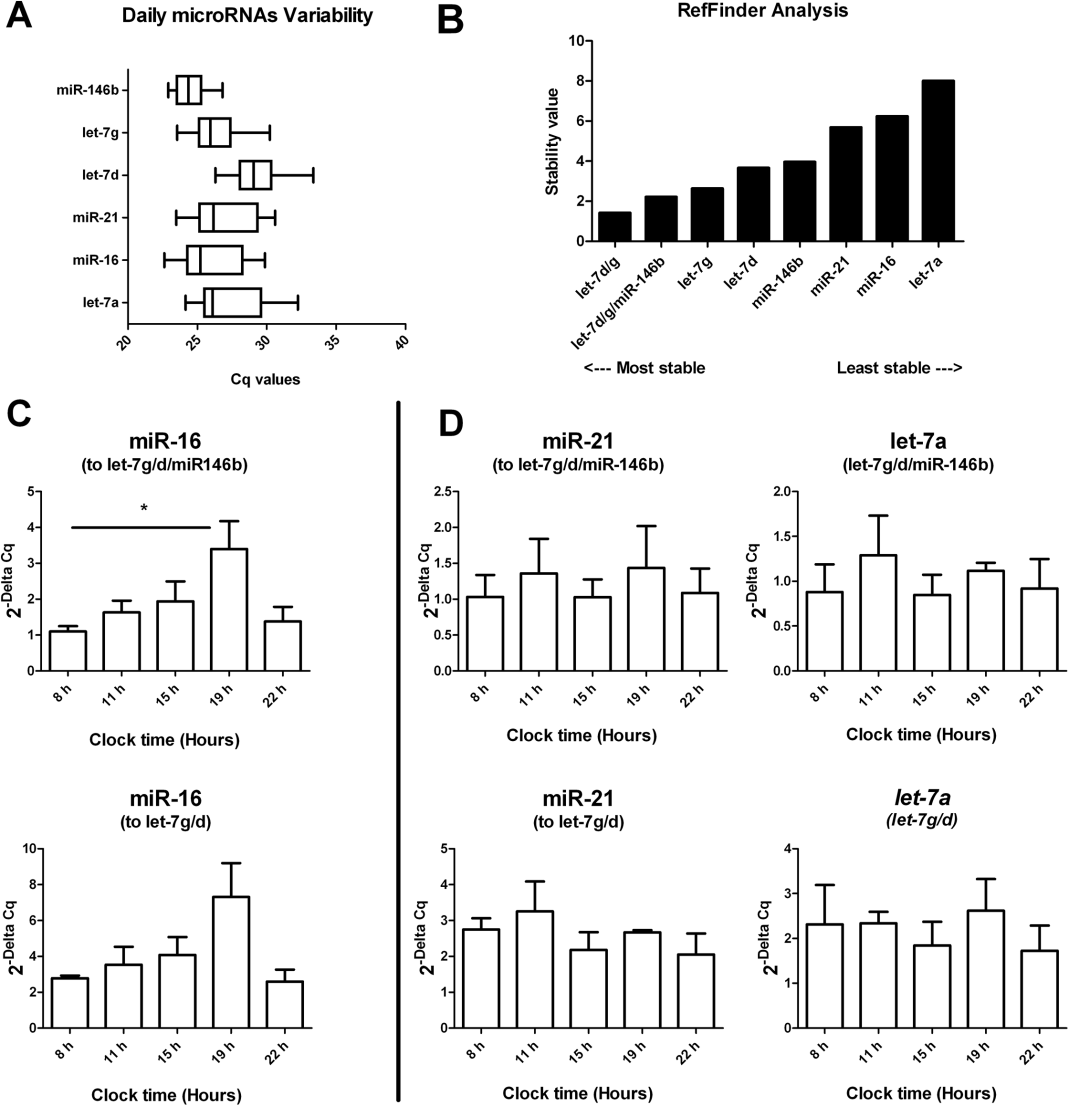
Analysis of daily miRNA fluctuations in whole milk. Endogenous references (let-7a, let-7g, let-7d, miR-16, miR-146b and miR-21) have been analyzed in milk samples collected from four mothers (A to D) and expressed at different times throughout a day. **A)** Box-and-whisker plots (1–99 percentile) showing raw Cq data of all measured miRNAs. **B)** RefFinder analysis sustains let-7g/d as the best combination for normalization followed by let-7g/d/miR-146b. **C)** MiR-16, using both normalization factors (let-7g/d/miR-146b and let-7g/d), exhibits daily fluctuations in the milk from four mothers (*p = 0*.*04*). Data are obtained from milk collected from four healthy donors during one day (*p<0.05). **D)** MiR-21 and let-7a appear relatively stable over 24 hours.

As shown Fig 4, box plots (1–99 percentiles) have been again used to visualize raw Cq variations of the six measured miRNAs. MiR-146b, let-7d and let-7g appeared relatively stable (Cq SDs respectively were 0.9, 1.5 and 1.6), while miR-16, miR-21 and let-7a were more variable (Cq SD ≥ 2; Fig 4A). Because choosing the most stably expressed reference gene for normalization is not always recommended [34], we further evaluated the candidate references by RefFinder. RefFinder analyses established that let-7d, let-7g and miR-146b were the most stable references, and that the geometric means of let-7d and let-7g (let-7d/let-7g) gave the best stability value, followed by the combination of the three controls let-7d/let-7g/miR-146b (Fig 4B). We have tested the two suggested combinations, let-7g/d and let-7g/d/miR-146b, in normalizing miR-21, miR-16 and let-7a levels. Both allowed identification of miR-16 fluctuations throughout the 24H (*p = 0*.*04*). Once again, the use of three references is preferred to the use of two or only one reference^27^. Using the normalization factor let-7g/d/miR-146b, the variance was reduced and the statistical efficiency improved compared to the use of let-7g/d. We discovered higher miR-16 levels in evening milk, collected from 18:00 to 20:00, than in morning milk, expressed from 7:00 to 9:00 (*p<0*.*05*) (Fig 4C). MiR-21 and let-7a levels, in both normalization strategies used, appeared relatively stable over the 24H (Fig 4D). As we could not possibly obtain a similar baseline time for all the donors, the four mothers differ in miR-16 fluctuations in their milk (S5 Fig); moreover, an intra-individual variability was found (S6 Fig).

We studied *in silico* if miR-16-5p had a predicted binding site targeting circadian core elements. Interestingly, it may target the 3'UTR of clock mRNA (p = 0.044). Using RNAhybrid [31] we got the *minimum free energy of hybridization* (*mfe*) of the miRNA-RNA duplexes, which only represents a fraction of the possible and existing structures. The *mfe* calculated for miR-16-5p with CLOCK mRNA was -21.8 kcal/mol, (S7 Fig).

## Discussion

Our study provides miRNA analyses by qPCR on human breast milk expressed by mothers of premature babies. Breast milk of mothers with preterm or term infants differ according to data on metabolome [35] or fatty acid composition [36]. Likewise, bioactive factors, such as IGF-I, TGF-ß, EGF, leptin, ghrelin, and adiponectin, involved in gut differentiation, epithelial proliferation, anti-inflammatory action, and metabolism, are more highly expressed in the milk of mothers with pre-term babies [37]. We aimed to design a fast assay on raw milk to analyze the potential role of miRNAs in the circadian physiology of breast feeding. Because of the precious value of this biological sample, which is primarily needed for the baby, we managed to design a q-PCR using only 100 μl of raw milk. In our hospital, we can routinely obtain 300 μL, which means that miRNAs can be checked both on raw and milk fractions provided the purification can be adapted to low amount of milk. All milk samples (n = 55) processed for RNA extraction gave a high variability in RNA concentration (S2 Fig). These values were not correlated with our available parameters (stage of lactation and hour of milk collection; data not shown). In addition, we analyzed miRNA levels in fresh milk, within 20 minutes post-collection and in stored milk, after 24 hours and one month at -80°C (S3 Fig) to explore the effect of freezing on miRNAs recovery. No differences were found in our six endogenous miRNAs control. We believe that this observation may be explained by the degradation of unsheltered miRNAs by milk RNAses. Breast milk is in high demand for biological experiments and a lot of facilities may resort to freezing to create biocollection. Our assay allows the analysis of whole milk collected under such conditions in order to analyze low volume. However our method is not proposed for a detailed analysis of milk fractions. For instance, Alsaweed et al (2015) have screened 8 kits to optimize extraction of miRNAs from fresh milk fractions [38]. Our assay suffers from several limitations and cannot be used to study milk fractionation as whole milk was frozen prior to milk fractionation, extraction of miRNA and further analysis. Our sample preparation protocol may have impacted on the miRNA extraction that was done *post hoc*, and may have contributed to miRNA contamination between different milk fractions due to cell lysis as well as to unknown potential changes in the miRNA expression levels for each milk fraction.

We have identified suitable miRNA references for qPCR analysis in human milk. First of all, we explored the use of synthetic non-human (e.g., C. elegans) miRNAs and selected endogenous miRNAs to normalize qPCR data in milk. The use of synthetic non-human (e.g., C. elegans) miRNAs, named “spike-in”, provides a reference for normalization of the *technical* variability but do not correct for sample-to-sample variability and therefore they cannot be used to normalize for any *biological* variability [19]. Our results showed that spiking-in external RNAs is not the best choice and that we have to recommend the use of three reference miRNA: hsa-let-7d, hsa-let-7g, hsa-miR-146-5P.

Here we found miR-21 and miR-16 stably expressed within month 2 of lactation in raw pooled milk samples (Fig 1). Once we assayed lipids and skim milk fractions, miR-146b had a higher stability in both, more than miR-21 and miR-16 (Fig 2). In addition, our analyses in whole milk and its lipids and skim milk fractions permitted the observation of differences in raw miRNA Cq values (Fig 3). These differences in miRNAs distribution might reflect specific regulatory mechanisms for the two types of vesicles: milk fat globules [10, 11, 39] and the population of extracellular vesicles present in skim milk [9], both secreted by mammary epithelial cells during milk production. Our results on time series showed that the use of three reference miRNA: hsa-let-7d, hsa-let-7g, hsa-miR-146 gives the best normalization factor (Fig 4A and 4B).

We have identified clock time-dependent fluctuations of miR-16-5p in preterm breast milk (Fig 4C). Once again, the unique dynamic composition of breast milk has been highlighted; bioactive components including miRNAs may change within month 2 of lactation, in its lipids and skim milk fractions, over 24 hours.

Daily fluctuations in mRNAs have been already reported in term milk. Maningat et al., 2009 performed a transcriptomic analysis on lipids fractions and showed 1,029 genes significantly modulated across the day [40]. A cluster of genes is highly expressed in the evening until the early morning, while other genes are mainly expressed during the day and turned off later in the evening. Core circadian genes have been found to exhibit the expected rhythms in milk throughout the 24 hours. However, the authors have “synchronized” the donors by obtaining a similar baseline point for all mothers [40]. In contrast, our analysis in preterm milk is designed to open the possibility to recruit unsynchronized mothers in clinical trials to study miRNAs profile and changes over the course of 24 hours.

We found variability in miR-16 patterns between donors and within a woman (month 2 of lactation). However, previous publications described a high intra- and inter-individual variability of nutrients in human milk [41–43], but they are far to be understood. Some authors think that milk composition may reflect infant needs [12].

In the work of Pigati et al., miR-16 has been used as an EC in human breast milk [17]. They investigated the miRNAs released *in vitro*, using human mammary epithelial cells (HMECs) as a cellular model. MiR-16 and miR-21 levels are constantly released in the culture media of human mammary epithelial cells with levels reflecting the cellular abundance. MiR-16 is the most constantly released and, for that reason, the authors used it as an endogenous reference to normalize miRNAs levels in human milk. Our results are not in favor of using hsa-miR-16-5p as endogenous control in preterm milk.

Up to now, circadian changes of miR-16 have been reported in the intestinal crypts of adult rats [44]. The authors hypothesized that miRNAs can be involved in the regulation of the circadian intestinal rhythmicity. They discovered that miR-16 exhibited circadian rhythmicity in the intestinal crypts of adult rats and exerted anti-proliferative effects on intestinal cells by acting directly on five cell cycle regulators (Ccnd1, Ccnd2, Ccnd3, Ccne1 and Cdk6) [44]. In human, the gastrointestinal functions of digestion and absorption are circadian regulated, notably the proliferation of intestinal epithelium [45–47]. In preterm infants, we have shown that gastric exfoliation of epithelial cells follows a circadian rhythm [48]. We speculate that daily miR-16 fluctuations in breast milk may have a dual meaning: in part it may reflect the metabolism of the mammary gland during lactation; conversely, it might have a role in transmitting maternal rhythms to breast-fed infants.

We are aware that more investigations are required to establish which factors may affect miR-16 levels in breast milk. From this study, we propose that another link can be explored between gastrointestinal rhythms of the breastfed infant and the lactocrin circadian signaling which, in part, might be mediated by the anti-proliferative miR-16. The daily miR-16 fluctuations in breast milk might act in the gastrointestinal epithelium of the lactating infant helping to establish and/or fine-regulate gastrointestinal rhythmicity and to coordinate it with the maternal rhythmicity.

## Supporting Information

S1 Fig Standard Curve for spike-in (cel-lin-4-5p). (TIF)

S2 Fig RNA concentration distributions from raw milk samples. RNA extracted from whole milk samples gave concentrations ranging from 105 to 665 ng/μL (mean concentration = 313 ng/μL, SD = 110, CV% = 35, n = 55).
(TIF)

S3 Fig MiRNAs analyzed in fresh and stored milk. MiR-16, miR-21, let-7a, let-7g and let-7d have been measured in fresh (unstored) milk, after 24 hours and 1 month later (stored at -80°C). Graph shows mean and SEM of four independent experiments using two donors. No significant difference has been found between fresh and stored milk.
(TIF)

S4 Fig External RNAs are not a suitable reference for miRNA normalization. **A)** Plot of external spike-in cel-lin-4 Cq values against Cq values of internal miRNA controls (miR-16, miR-21 and let-7a) indicating no correlation. **B)** Positive correlation between RNA dilution factors used for making diluted RNA for the RT reaction and spike-in Cq values; negative correlation between RNA dilution factors and spike-in levels expressed as 2^-Cq^. **C)** Positive correlation between Cq values of two endogenous references (miR-16 and miR-21).
(TIF)

S5 Fig MiR-16, let-7a and miR-21 levels measured in the four mothers’ milk throughout clock time. (TIF)

S6 Fig MiR-16, let-7a and miR-21 levels measured in milk collected from one donor during two different days of lactation. (TIF)

S7 Fig MiRNA-RNA duplex: miR-16-5p-3’UTR mRNA CLOCK. (TIF)
