# Supplementary figures and images for: MiRNA Analysis by Quantitative PCR in Preterm Human Breast Milk Reveals Daily Fluctuations of hsa-miR-16-5p
Source: PLoS One. 2015 Oct 16;10(10):e0140488. doi: 10.1371/journal.pone.0140488 (PMC4608744; doi:10.1371/journal.pone.0140488)

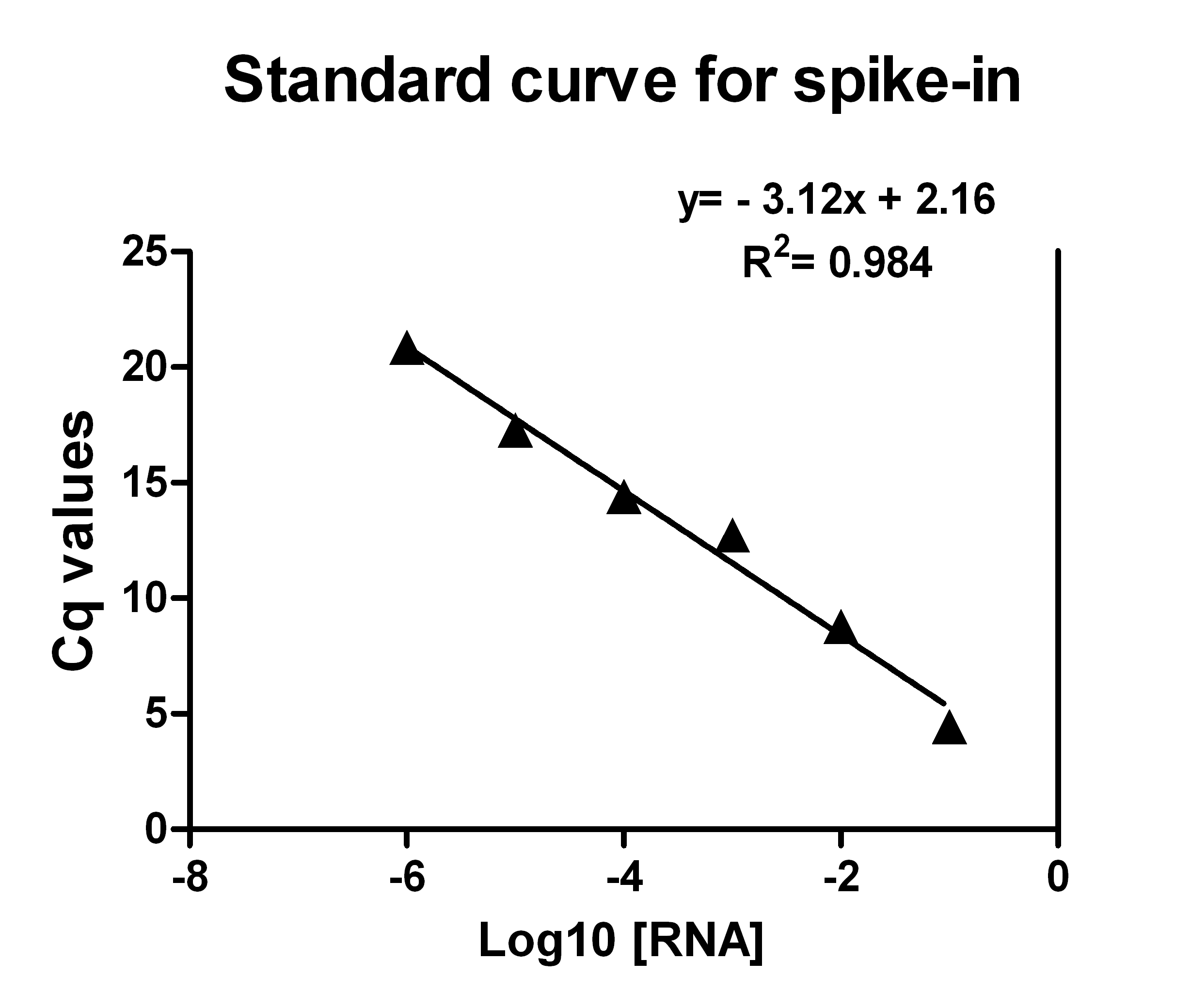

Supplement: S1 Fig — (TIF) [file pone.0140488.s001.tif]

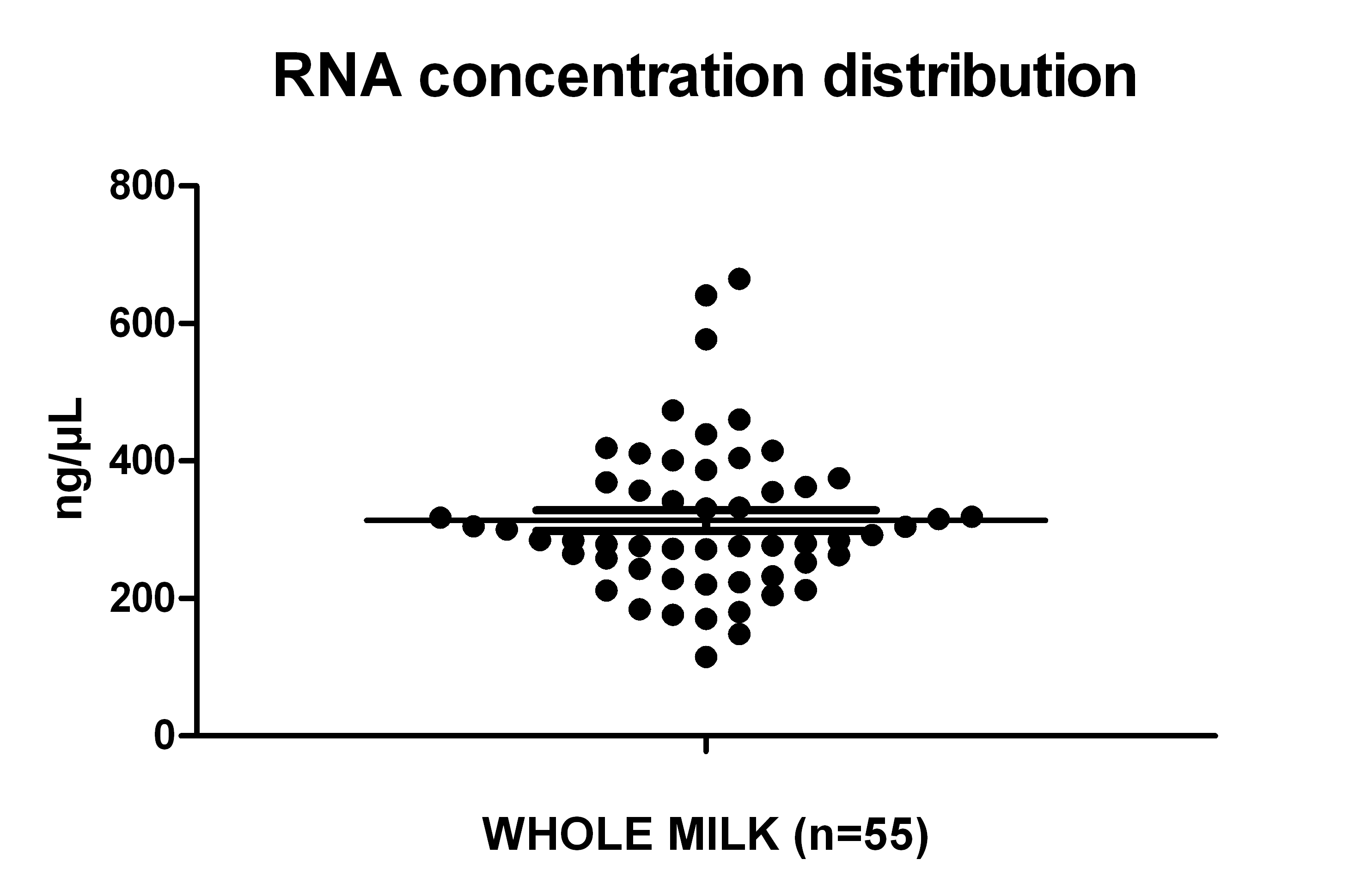

Supplement: S2 Fig — RNA extracted from whole milk samples gave concentrations ranging from 105 to 665 ng/μL (mean concentration = 313 ng/μL, SD = 110, CV% = 35, n = 55). (TIF) [file pone.0140488.s002.tif]

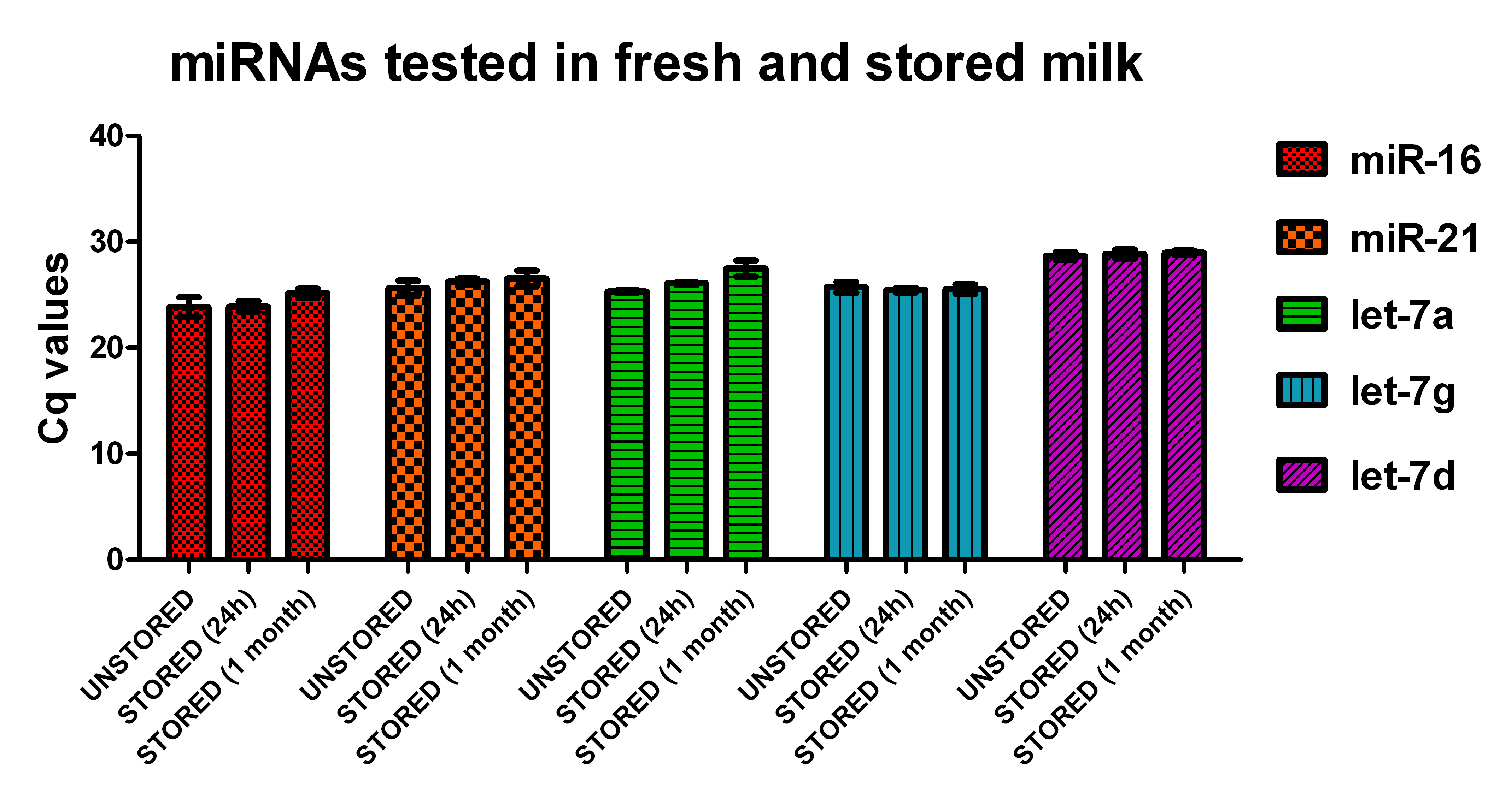

Supplement: S3 Fig — MiR-16, miR-21, let-7a, let-7g and let-7d have been measured in fresh (unstored) milk, after 24 hours and 1 month later (stored at -80°C). Graph shows mean and SEM of four independent experiments using two donors. No significant difference has been found between fresh and stored milk. (TIF) [file pone.0140488.s003.tif]

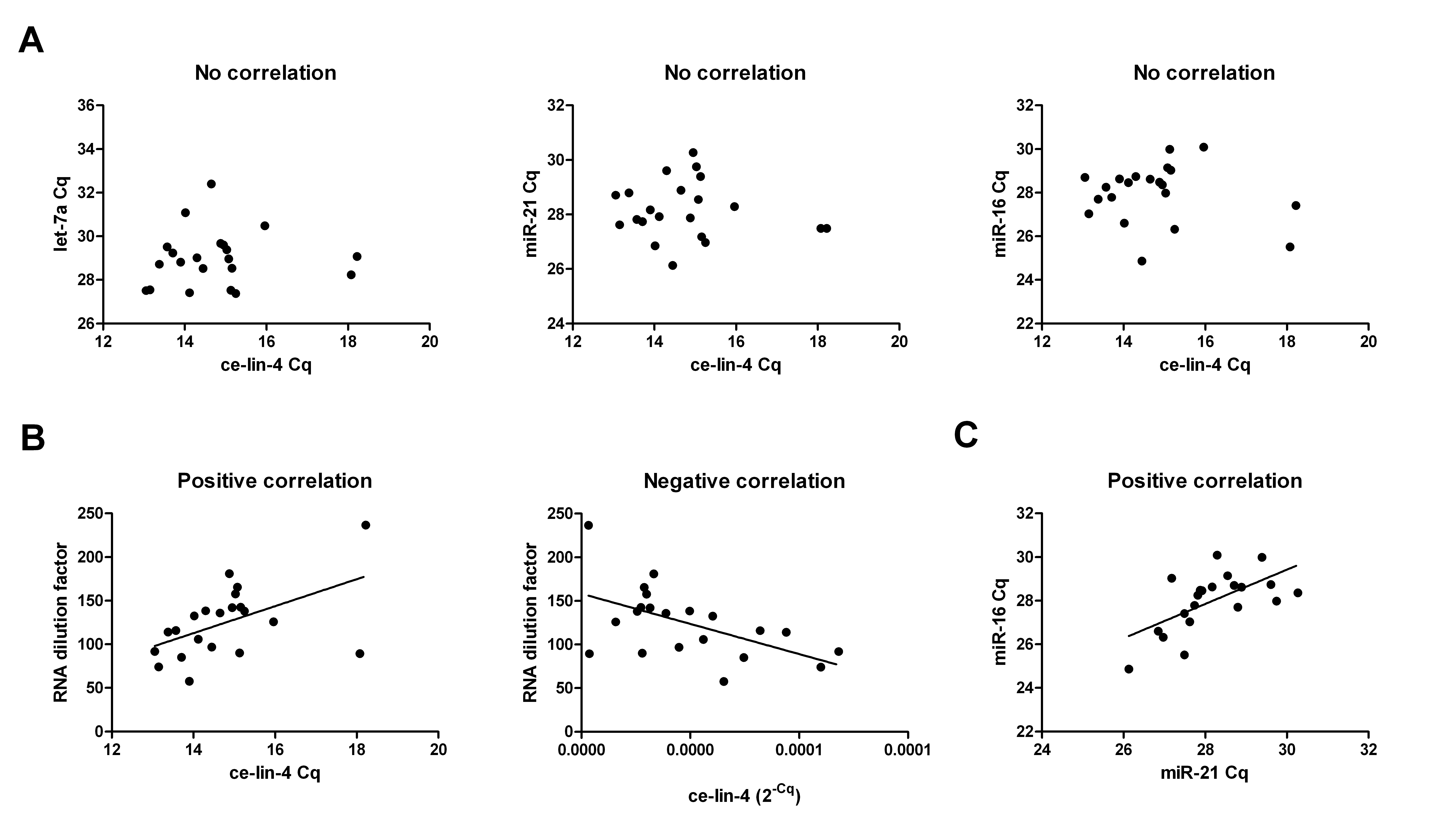

Supplement: S4 Fig — A) Plot of external spike-in cel-lin-4 Cq values against Cq values of internal miRNA controls (miR-16, miR-21 and let-7a) indicating no correlation. B) Positive correlation between RNA dilution factors used for making diluted RNA for the RT reaction and spike-in Cq values; negative correlation between RNA dilution factors and spike-in levels expressed as 2-Cq. C) Positive correlation between Cq values of two endogenous references (miR-16 and miR-21). (TIF) [file pone.0140488.s004.tif]

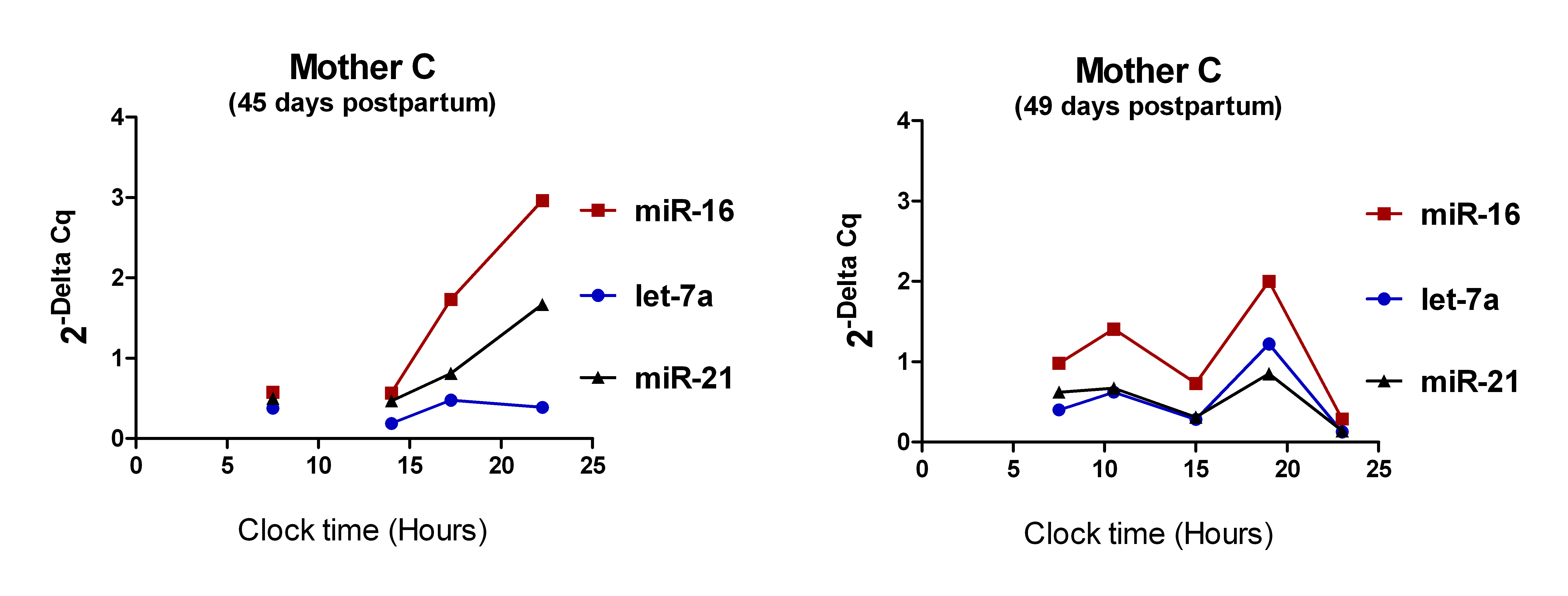

Supplement: S6 Fig — (TIF) [file pone.0140488.s006.tif]

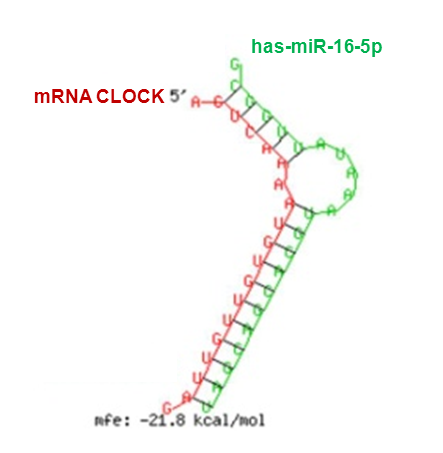

Supplement: S7 Fig — (TIF) [file pone.0140488.s007.tif]
